# Effect of Single Administration of Mulberry Milk on the Cognitive Function of 6–12-Year-Old Children: Results from a Randomized, Placebo-Controlled, Crossover Study

**DOI:** 10.1155/2020/6123759

**Published:** 2020-06-27

**Authors:** Wipawee Thukham-mee, Jintanporn Wattanathorn, Woranan Kirisattayakul, Panakaporn Wannanon

**Affiliations:** ^1^Integrative Complementary Alternative Medicine Research Center in Research Institute for Human High Performance and Health Promotion, Khon Kaen University, Khon Kaen 40002, Thailand; ^2^Department of Physiology, Faculty of Medicine, Khon Kaen University, Khon Kaen 40002, Thailand

## Abstract

Currently, cognitive enhancers are considered necessary because they play a critical role in daily and social behaviors. The cognitive-enhancing effect of mulberry milk has gained attention due to the cognitive-enhancing effect of this anthocyanin-rich substance and the cognitive-enhancing effect of mulberry fruit in animal models. However, the effect of anthocyanin-rich mulberry milk in clinical trials especially in children is still unknown. This study was a randomized double-blind crossover intervention. A total of forty-six healthy, normal, cognitive subjects aged 6–12 years old were provided mulberry milk (containing mulberry 10 g) or placebo milk (50 mL). Attention and cognitive function were assessed using the auditory odd ball paradigm of event-related potential, whereas working memory was assessed using a computerized battery test. The assessment was performed at baseline and then at 1.5 and 3 hours postdosing. At the end of study period, the activities of acetylcholinesterase (AChE) and monoamine oxidase (MAO) together with that of saliva cortisol were determined. Following mulberry milk intervention, the decreased N100 latency and the increased P300 amplitude were increased both at 1.5 and 3 hours after dosing. The decreased response time of digit updating was observed both at 1.5 and 3 hours after dosing, whereas the decreased response time of picture updating was observed at 3 hours after dosing. In addition, the reduction of saliva cortisol was also observed at both periods. The improvement of attention and cognitive processing capabilities together with the working memory suggests the cognitive-enhancing potential of mulberry milk for school-age children. The possible underlying mechanism may be associated partly with the reduction of cortisol, a stress hormone.

## 1. Introduction

Cognitive performance, a group of functions that includes memory, general intelligence, learning, language, orientation, perception, attention, concentration, and judgment, is regarded as an important factor of children's academic achievement [1–2]. In addition, it also indicates the growth of a child's ability to think and reason or executive function capacity. This capacity serves a critical role in daily and social behaviors. The development of this capacity is reported to occur throughout childhood and adolescence periods and is parallel to the developmental changes that occur in the brain throughout the mentioned periods [3].

Cognitive capabilities and their developments are under the influence of many factors, including dietary factors [4]. The strong association between nutritional intake and neurocognitive development in childhood has been reported [5]. Accumulative lines of evidence have demonstrated that flavonoids, a class of polyphenols found in abundance in vegetables and fruits, can improve cognitive performance across the age groups after a single and repeated administration [6–7]. Several clinical data clearly reveal that anthocyanins, a subclass of flavonoids, significantly enhance cognitive function [8–10]. This raises the possibility that an anthocyanin-rich substance can be used as a functional ingredient in food and drinks in order to produce functional food.

The ripe fruits of mulberry (*Morus alba*), a plant in the family of Moraceae, is also rich in anthocyanins [11]. They have been consumed for a long time in various regions of Asia, including Thailand. *In vitro* data obtained from a previous study demonstrated that mulberry fruit extract possesses higher contents of polyphenolic compounds and flavonoids (especially anthocyanins) than blueberry (*Vaccinium corymbosum* L.) It also exhibited a more potent antioxidant activity [12]. Preclinical data showed that anthocyanin-rich mulberry extract possessed a cognitive-enhancing effect and could improve memory impairment in vascular dementia following ischemic stroke [12]. In addition, it also enhanced cognitive function in an animal model of menopause with metabolic syndrome. Therefore, we hypothesized that anthocyanin-rich functional drinks from mulberry fruit should be able to enhance cognitive function in children. To date, no clinical data supporting this hypothesis are available. Therefore, a clinical trial concerning the aforementioned issue is required. Since the Thai government policy promoted the daily milk consumption for health in children, we aimed to evaluate the effect of mulberry milk on attention and cognitive processing ability together with working memory in children aged between 6 and 12 years old.

## 2. Materials and Methods

### 2.1. Ethical Statement and Trial Registration

This study was performed according to the Declaration of Helsinki (ethical principles for research involving human subjects). The protocol was under the approval of the Institute's Human Ethical Committee under HE611295. This project was also registered online as part of the Thai Clinical Trial Registry (TCTR20190725005).

### 2.2. Participants

A total of 50 student volunteers aged between 6 and 12 years old were recruited to participate in this study. However, only 46 were able to participate throughout the whole study period. Four persons withdrew due to inconvenient transportation. The parents or legal guardians of all the children who participated in this study provided written consent forms. All participants used Thai language as the primary language, and their healthy conditions were confirmed via physical examination by the physician of the project. In addition, no head injury and milk allergy were presented.

### 2.3. Protocol

**Figure 1:**
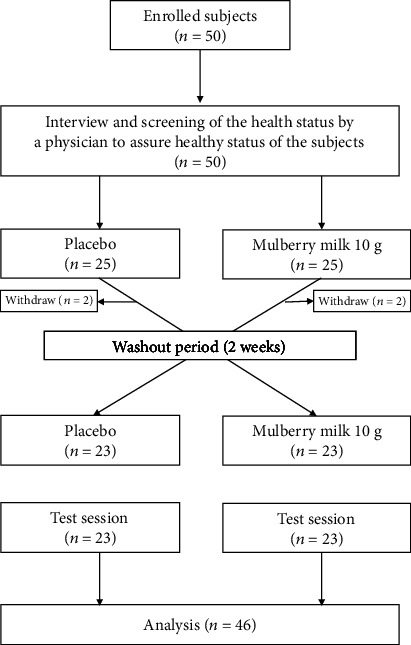
Schematic diagram showing intervention allocation and participants in the study.

To explore the potential cognitive-enhancing effect of mulberry milk, a placebo-controlled crossover trial was conducted. The participants were randomly assigned to receive one serving of placebo or 10 g mulberry milk per person. Each serving was 50 mL and contained the polyphenolic compounds around 469 ± 3.48 mg GAE/g sample. The contents of flavonoids and anthocyanins were 211.64 ± 0.31 mg quercetin/g sample and 6.26 ± 0.62 g cyanin-3-glucoside equivalent/g sample, respectively. In addition, it also contained cyanidine-3-glucoside (C3G), cyanidine-3-rutincoside (C3R), rutin, and quercetin at the concentrations of around 0.25-0.45, 0.25-0.64, 0.50-0.99, and 0.02-0.025 mg/10 g sample. The placebo contained pasteurized milk (31.84 g), maltodextrin (4.46 g), and sugar (0.7 g), whereas the mulberry milk contained the aforementioned substances and mulberry powder (1.76 g). The color and berry flavor were also added to the placebo. Therefore, both the placebo and mulberry milk have the same appearance. All subjects were requested to maintain their normal eating habits and exercise habits. In addition, all subjects must drink mulberry milk within 5 minutes. The cognitive function of each subject was determined using the event-related potential and computerized battery test at baseline level or prior to milk consumption and at 1.5 and 3 hours after milk consumption. The level of cortisol and the activities of acetylcholinesterase and total monoamine oxidase in saliva were also monitored. After a 2-week washout period, all participants must complete both arms of a trial. The schematic diagram that illustrated the protocol used in this study was shown in Figure 1.

### 2.4. Assessment of Cognitive Function

The cognitive function was measured both by event-related potential via the auditory odd ball paradigm and by the cognitive battery test of psychometric and psychological tests via a computer.

#### 2.4.1. Cognitive Battery Test

The cognitive battery test of psychometric and psychological tests used in this study comprised of 10 memory tasks including digit and memory updating, flank arrow, left or right, up and down, switch up-down-left-right, odd even, vowel consonant, switch letter number, and Stroop Thai tests. They were used for monitoring updating, shifting, and inhibition capacities of working memory in this study.

*(1) Digit Updating*. Subjects were exposed to four sets of a digit task, and subjects were asked to memorize the last digit. According to this test, 4 lists of digits were presented to the subject one at a time and the subject was requested to memorize the last digit of each set. The subject must recall them when they were asked to produce the last 4 digits.

*(2) Picture Updating*. Three sets of pictures were presented to the subject. The subject was asked to maintain the information of the last picture of the first list and the first and second pictures back from the last picture of the second and third lists of pictures, respectively.

*(3) Flank Arrow*. According to this test, the subjects must respond to a target stimulus (→, ←). They were requested to press “1” when the head of the arrows were in the same direction and press “2” when the head of the arrows were not in the same direction.

*(4) Left-Right*. The stimuli “←↓” and “→↓” were presented to the subjects. The subjects were asked to focus on the arrow with the arrowheads on the left or right direction. They must press “1” when the arrowheads pointed to the left direction and must press “2” when the arrowheads pointed to the right direction.

*(5) Up-Down*. The stimuli “←↓” and “→↓” were presented to the subjects. The subjects were asked to focus on the arrow with the arrowheads on the up or down direction. They must press “1” when the arrowheads pointed downward and must press “2” when the arrowheads pointed upward.

*(6) Switch Up-Down-Left-Right*. After the exposure to the stimuli “←↓” and “→↓,” the subjects were requested to focus on the direction of the presented arrow (up or down) when the stimuli presented at the top of the screen. They must press “1” when the arrowheads pointed downward and must press “2” when the arrowheads pointed upward. In addition, when the stimuli presented at the bottom of the screen, they must press “1” when the arrowheads pointed to the left direction and must press “2” when the arrowheads pointed to the right direction.

*(7) Odd Even*. The digits together with alphabets consisting of consonants and vowels were provided to the subjects. They were requested to focus on the digit and press “1” when the digit was odd and press “2” when the digit was even.

*(8) Vowel Consonant*. The subjects were provided the same stimuli as mentioned in the odd even task. In this test, the subjects must focus on the alphabet. They were requested to press “1” when the alphabet was a consonant and press “2” when the alphabet was a vowel.

*(9) Switch Letter Number*. The subjects were provided stimuli as mentioned in the odd even and vowel consonant tasks. When the stimuli were provided at the top of the screen, the subjects must focus on the number. They must press “1” when the digit was odd and press “2” when the digit was even. They must focus on the alphabet when the stimuli was presented at the bottom of the screen. They must press “1” when the alphabet was a consonant and press “2” when the alphabet was a vowel.

*(10) Stroop Color-Word Test*. A series of colored words written in colored ink was presented to the subject. The subject was requested to press “1” when the presented letter matched with the color and press “2” when the letter did not match with the color.

The accuracy and response time of each test were recorded and expressed as percent of accuracy and millisecond.

#### 2.4.2. Event Related Potential (ERP) Recording Procedure

The brainwave data were recorded from Ag-AgCl disc electrodes according to the international 10/20 system with reference to linked earlobes using a 40-channel electrode cap (Neuroscan, Inc., Sterling, USA). The resistance of the electrode was less than 10 k*Ω*. Two different tones of auditory stimuli at 60 dB and the frequencies of 650 Hz and 1 kHz were provided to the subjects. The low frequency was regarded as the standard stimuli (nontarget tone), whereas the high frequency was regarded as targeted tone. The target tone and nontarget tone were provided at 15% and 85% of total stimuli, respectively. The interstimulus interval was 1250 msec. Participants were informed to pay attention and listen to the tones of stimuli through the headphone and respond to each tone by pressing the response button in front of them. The total recording time was 10 minutes.

Data obtained from the Fz and Cz locations were used for the analysis based on the previous information that these areas showed the optimum peak patterns of N100 and P300 [13]. The analysis was performed using Scan 4.3 analysis software (Neuroscan, Inc., Sterling, USA). All artifacts and ocular artifacts were removed from the continuous EEG prior to the extraction of ERP waves. The epochs were extracted from the EEG-free artifact from 100 msec prestimulus and continued to 500 msec poststimulus. The baseline correction was also applied to each epoch. Any changes of voltage below 0.1 *μ*V or above 50 *μ*V were rejected from further analysis. The negative peak that presented between 65 and 135 msec was defined as N100, while the positive peak that presented between 280 and 375 nm was defined as P300 [14]. Both latencies and amplitudes of both brainwaves were recorded and analyzed.

### 2.5. Biochemical Assessments

#### 2.5.1. Determination of Acetylcholinesterase (AChE) Activity

The determination of the acetylcholinesterase (AChE) activity of mulberry milk was performed using the Ellman method [15]. Briefly, all of the following substances were mixed in 96-well plates: 10 *μ*L of control or saliva, 10 *μ*L of 0.2 mM DTNB, 20 *μ*l of 0.1 mM PBS (pH 8.0), and 10 *μ*L of 15 mM ATCI. After incubation at room temperature for 5 minutes, an absorbance was measured at 415 nm. AChE activity was calculated as follows:

(1) $$\begin{array}{c} \text{AChE activity}=\left(\frac{∆A}{1.36\times 10^{4}}\right)\times \frac{1}{\left(20/230\right)}C, \end{array}$$

where *∆A* is the difference of absorbance/minute and *C* is the protein concentration of the brain homogenate.

#### 2.5.2. Determination of Monoamine Oxidase Activity

Monoamine oxidase (MAO) activity was determined according to the modified method of Holt et al. [16]. Briefly, 50 *μ*L of saliva was mixed with chromogenic solution which consisted of 1 mM vanillic acid, 500 *μ*M 4-aminoantipyrine, and 4 U·mL^−1^ peroxidase in 0.2 M potassium phosphate buffer, pH 7.6. Then, tyramine was added and used as a substrate. After incubation for 30 minutes at 37°C, the absorbance was measured at 490 nm with a microplate reader (iMark™ Microplate Absorbance Reader). The data was expressed as nmol/h/mg protein.

#### 2.5.3. Determination of Cortisol

The determination of saliva cortisol was performed using an ELISA kit. In brief, an aliquot of 25 *μ*L of standards or saliva was mixed with 200 *μ*L of cortisol-HRP conjugate solution and subjected to a 60-minute incubation period at 37°C. Following this process, washing was performed with buffer for three times, and TMB (tetramethylbenzidine) substrate solution at a volume of 100 *μ*L was added and incubated at room temperature for 15 minutes. At the end of the incubation period, a stop solution was added and an absorbance at 450 nm was recorded via a microplate reader. Cortisol was used as a standard reference. The results were expressed as ng/mL [17].

### 2.6. Statistical Analysis

Data analysis was performed on a per-protocol analysis. Comparisons between groups regarding cognitive function, the level of saliva cortisol, and the activities of AChE and MAO in saliva were performed using an unpaired Student's *t*-test. The level of significance was regarded at *p* value < 0.05.

## 3. Results

### 3.1. Effect of Mulberry Milk on Cognitive Function

At baseline, no significant differences in any data of the placebo- and mulberry milk-treated groups were observed as shown in Table 1. In addition, Table 2 demonstrated that no significant differences in both latencies and amplitudes of both N100 and P300 waves recorded at both the Fz and Cz locations were observed between the placebo- and mulberry milk-treated groups at baseline level. At 1.5 hours after the administration of mulberry milk, the latencies of N100 both at the Cz and Fz locations decreased (*p* value < 0.01 for all, compared to the placebo-treated group). However, at 3 hours after the intervention, a significant reduction of the N100 latency was observed only at the Cz location (*p* value < 0.05; compared to the placebo-treated group). In addition, the increase in P300 amplitude was also observed at the aforementioned location (*p* value < 0.05 for all, compared to the placebo-treated group) as shown in Table 2.

Table 3 shows the effect of mulberry milk on working memory assessed by the computerized battery test. No significant changes of any parameters were observed between the placebo- and mulberry milk-treated groups at baseline level. It was found that the children who consumed mulberry milk showed a reduction in response time in the digit updating test both at 1.5 and 3 hours after the administration of mulberry milk (*p* value < 0.05, compared to the placebo group). In addition, a decreased response time in the picture updating test was also observed in the mulberry-treated group 3 hours after the administration of mulberry milk (*p* value < 0.01, compared to the placebo group).

### 3.2. Effect of Mulberry Milk on Biochemical Changes

Table 4 shows the effects of mulberry milk on various biochemical changes including the level of cortisol and the activities of AChE and total MAO in saliva. It was found that at baseline level, no significant changes in the aforementioned parameters were observed. Interestingly, a significant reduction of saliva cortisol was observed at both 1.5 and 3 hours after the intervention (*p* value < 0.001 and *p* value < 0.05, compared to the placebo group), whereas no significant changes in other parameters were observed at both time window periods.

## 4. Discussion

The ability to retain task-relevant information in an accessible state over time (working memory) and the ability to selectively process information in the environment (attention), which are recognized as critical aspects of our cognitive capacities, play the pivotol roles in the capacity to perform some complex tasks [18] including the academic performance of children. Therefore, cognitive enhancers have gained much attention.

At present, working memory, a valid indicator that indicates cognitive function in children [19], can be assessed using event-related potential (ERP) [20]. This measurement is the assessment of the electrical potential or brainwaves caused by how the brain structures respond to specific events or stimuli. This type of brainwave can be stimulated by various stimuli including sensory, motor, and cognition events. The obtained brain waves are expressed as average values of brainwaves at intervals related to various events. The components of the brainwaves are both positive (P) and negative (N) deflections. The brainwaves that often occur when stimuli appear are N1 or N100 waves which usually occur at around 90-200 milliseconds after the stimulation. The brain wave which plays a critical role on cognitive processing or executive function or P3 or P300 often occurs at 250-400 milliseconds after the stimulation. The latter wave is often found while the brain is distinguishing one event from another. Therefore, it is used as an indication of cognitive processing [21]. Both attention and cognitive processing are reported to be associated with the alterations of neurotransmitters. It has been shown that acetylcholine (ACh) plays a crucial role in both cognitive processing and sustained attention [22–23]. In addition, norepinephrine (NE) and dopamine (DA) also play an essential role in attention [24–26]. It has been revealed that ACh exerts great influence on the change of N100 and P300, whereas NE and DA exert influence only on P300 [27–29]. Unfortunately, our data failed to produce the significant changes of AChE and MAO in saliva. The possible explanation for the lack of significant change of AChE might occur partly because the change of saliva AChE does not represent the changes of AChE and the cholinergic system in the target tissue due to the weak correlation of AChE in saliva and AChE in other tissues such as erythrocytes [30]. However, the recent study has reported that saliva AChE can be used as potential biomarkers to indicate the significant impairment of the cholinergic system and memory impairment in patients with Alzheimer's disease [31]. Therefore, the lack of significant change in saliva AChE might occur partly because cholinergic system change may not play a crucial role in the memory-enhancing effect induced by a single dose of mulberry milk. Based on the information that the saliva MAO shows the relationship with the recognition memory capability [32], its change was assessed to explore the role of the cognitive-enhancing effect of mulberry milk in this study. The lack of significant change of this parameter suggests that the MAO change, which gives rise to the alteration in the monoaminergic system including dopamine function, may not play a role on the cognitive-enhancing effect of mulberry milk after a single administration.

The working memory is also under the influence of stress. It has been reported that the reduction of saliva cortisol is associated with enhanced short-term memory [33] and attention [34]. Based on these pieces of information, we also investigate the effect of mulberry milk on the changes of the aforementioned parameters. Our data have clearly shown the significant reduction of saliva cortisol together with the reduction of N100 latency and the increase in P300 amplitude, which indicate the improvement of attention and cognitive processing of working memory. Previous findings have shown that the proper level of cortisol can enhance consolidation and long-term potentiation giving rise to the improvement of memory, while too low or too high levels of cortisol impair working memory [35]. In addition, it has been demonstrated that the rapid nongenomic effect of cortisol can increase emotional interference which in turn decreases selective attention capacity [36].

Since the central executive function is the most important component of working memory, we also investigated the effect of mulberry on the central executive function component using the computerized battery test. According to this test, there are 3 main components of executive function including response inhibition, updating and monitoring of working memory, and mental set shifting. “Inhibition” was measured by focusing on the ability to suppress the influence of interfering information such as a Stroop test [37]. It has been shown that “Updating Working Memory” represents our cognitive capacity for simultaneous processing of multiple tasks. It is a screening and coding system that reviews information based on its circumstantial significance, constantly eliminating extraneous information and replacing it with more relevant information [37]. This capacity can be assessed using digit and picture updating tests [38]. In addition to the aforementioned capabilities, the ability to adapt dynamically to changing task demands and contexts or “Shifting,” which involves back and forth movements between tasks and higher and lower levels of mental processing, also plays a critical role on the executive function compartment of working memory [39]. This can be assessed by a number-letter shifting test. The data obtained from this study reveal that mulberry milk can improve both digit and picture updating capacity. Based on the previous information that the updating capacity and N100 are under the influence of attention [40], we do suggest that mulberry milk improves attention efficiency manifesting as a reduction of N100 which in turn increases the updating capacity of working memory manifesting as the decrease in response times of both digit and picture updating tests. The previous study has demonstrated that P300 is markedly shown at the central and posterior electrodes, and the origins are associated with the inferior parietal lobe (temporoparietal junction), supplementary motor cortex, anterior cingulate, and inferior and superior temporal gyrus, as well as the insula and hippocampus, in addition to the lateral prefrontal cortex [41]. Therefore, mulberry milk may possibly improve the function of the neuronal network, which plays a critical role on attention and then spreading the activity more posteriorly to the inferior parietal lobe mentioned earlier giving rise to the improvement of the response time of N100 and P300 in the Cz location together with the improvement of the updating working memory. This corresponds with the report that food quality and good nutrition are associated with cognitive function especially with the working memory of children [42–44]. It has been shown that at Fz, no significant change of P300 was observed because of numerous associated structures located in the more central and posterior parts. Therefore, the increase in P300 amplitude is observed only at the Cz location.

A pile of evidence has demonstrated that flavonoids exert profound health benefits including a cognitive-enhancing effect. Foods which are rich in anthocyanins, a subclass of flavonoids that includes blueberry and black currant, reveal a cognitive-enhancing effect after a single-dose administration [9, 11]. These findings correspond with our study which show the cognitive-enhancing effect of mulberry milk which is rich in anthocyanins at 1.5 and 3 hours after consumption. Taking all data together, we suggest that the anthocyanins in mulberry milk may partly have a role in the cognitive-enhancing effect observed in this study.

The limitation of this study is the inability to collect blood from the children with no clinical indication due to the invasive technique of blood collection induced by venopuncture. However, the level of saliva AChE may not represent the actual change of AChE in the brain and serum. Further researches for the alterations of the aforementioned changes in the brain using positron emission tomography (PET) is required and can provide the precision changes of the mentioned substances. The studies which show the close relationship between the changes of both substances in target tissue and in serum together with the relationship of the mentioned substances in target tissue and in saliva are also required to confirm that the salivary changes of the substances can be used as the validated biomarkers to indicate the changes of the mentioned substances in the target organ.

## 5. Conclusion

This study is the first study which demonstrates the cognitive-enhancing effect of anthocyanin-rich mulberry milk after a single administration. The possible underlying mechanism may be associated with the reduction of stress hormone and cortisol, which can improve attention and the updating mechanism of working memory. Therefore, it can be a candidate food supplement for school-age children at the ages between 6 and 12 years old. However, more research with more subjects are required to assure the cognitive-enhancing effect for most of the population.

The current data have clearly demonstrated the cognitive-enhancing effect of mulberry milk. Mulberry milk produces a significant decrease in N100 latency and response time in digit and picture updating tests but an increase in P300 amplitude together with a decrease in saliva cortisol.

## Figures and Tables

**Table 1:** The demographic data of subjects. Data are presented as mean ± SEM and compared between groups (n = 46/group).

Characteristics	Placebo	Mulberry milk 10 g	*p* value
Age (year)	8.87 ± 0.35	8.83 ± 0.38	0.933
Gender (male/female)	20/26	20/26	—
Pulse rate (beats/min)	105.86 ± 3.74	97.39 ± 2.21	0.053
Systolic blood pressure (mmHg)	74.83 ± 4.01	67.52 ± 4.82	0.250
Diastolic blood pressure (mmHg)	85.39 ± 4.50	84.57 ± 3.69	0.888
Body weight (kg)	40.53 ± 4.24	30.98 ± 2.30	0.054
Body height (cm)	136.43 ± 3.28	132.96 ± 2.99	0.437
Body mass index (BMI)	19.39 ± 1.06	16.99 ± 0.66	0.058

**Table 2:** The effect of mulberry milk on event-related potential (ERP) (*n* = 46/group). ^∗^*p* value < 0.05 and ^∗∗^*p* value < 0.01, respectively, compared to the placebo group.

Location	Wave	Treatment group	Baseline	*T* 1.5 h	*T* 3 h
Fz	N100 latency (ms)	Placebo	99.31 ± 3.26	108.97 ± 1.96	104.40 ± 4.19
		Mulberry milk 10 g	103.17 ± 2.86 *p* value = 0.37	98.36 ± 2.66 *p* value = 0.002^∗∗^	95.20 ± 2.97 *p* value = 0.078
	N100 amplitude (*μ*V)	Placebo	13.96 ± 1.33	12.66 ± 1.13	15.83 ± 1.25
		Mulberry milk 10 g	16.13 ± 1.34 *p* value = 0.256	14.32 ± 1.59 *p* value = 0.400	14.88 ± 1.04 *p* value = 0.561
	P300 latency (ms)	Placebo	329.60 ± 2.91	329.31 ± 4.57	320.72 ± 3.10
		Mulberry milk 10 g	326.50 ± 5.07 *p* value = 0.599	325.81 ± 4.35 *p* value = 0.581	320.97 ± 4.28 *p* value = 0.962
	P300 amplitude (*μ*V)	Placebo	34.04 ± 2.02	32.14 ± 1.65	28.68 ± 2.04
		Mulberry milk 10 g	31.96 ± 2.11 *p* value = 0.480	34.81 ± 1.86 *p* value = 0.285	33.60 ± 1.74 *p* value = 0.070

Cz	N100 latency (ms)	Placebo	100.64 ± 2.89	109.07 ± 1.95	109.03 ± 2.72
		Mulberry milk 10 g	105.50 ± 2.49 *p* value = 0.205	98.39 ± 3.27 *p* value = 0.008^∗∗^	99.39 ± 3.27 *p* value = 0.029^∗^
	N100 amplitude (*μ*V)	Placebo	15.54 ± 1.76	15.74 ± 2.21	17.54 ± 1.69
		Mulberry milk 10 g	16.73 ± 1.54 *p* value = 0.611	13.91 ± 1.45 *p* value = 0.482	17.06 ± 1.73 *p* value = 0.843
	P300 latency (ms)	Placebo	324.82 ± 4.21	320.97 ± 4.85	319.36 ± 3.39
		Mulberry milk 10 g	324.61 ± 5.00 *p* value = 0.974	326.72 ± 3.88 *p* value = 0.357	321.30 ± 4.17 *p* value = 0.720
	P300 amplitude (*μ*V)	Placebo	37.40 ± 2.13	30.35 ± 1.71	27.59 ± 1.94
		Mulberry milk 10 g	31.25 ± 3.00 *p* value = 0.099	36.11 ± 1.91 *p* value = 0.028^∗^	33.93 ± 2.21 *p* value = 0.035^∗^

**Table 3:** The effect of mulberry milk on the cognitive battery test (*n* = 46/group). ^∗^*p* value < 0.05 and ^∗∗^*p* value < 0.01, respectively, compared to the placebo group.

Test items	Group	Baseline	*T* 1.5 h	*T* 3 h
Digit updating				
Accuracy (%)	Placebo	67.63 ± 3.48	61.62 ± 4.75	63.84 ± 3.86
	Mulberry milk 10 g	70.37 ± 3.11 *p* value = 0.559	62.20 ± 2.67 *p* value = 0.910	61.14 ± 3.62 *p* value = 0.611
Response time (ms)	Placebo	2724.81 ± 224.10	2825.62 ± 229.78	2906.81 ± 265.29
	Mulberry milk 10 g	3416.83 ± 598.83 *p* value = 0.282	2158.74 ± 105.29 *p* value = 0.002^∗∗^	2179.67 ± 139.34 *p* value = 0.017^∗^

Picture updating (0 back)				
Accuracy (%)	Placebo	60.07 ± 4.04	62.39 ± 4.83	59.88 ± 3.84
	Mulberry milk 10 g	58.02 ± 4.53 *p* value = 0.737	60.96 ± 2.64 *p* value = 0.780	59.76 ± 3.88 *p* value = 0.983
Response time (ms)	Placebo	1056.17 ± 91.93	870.12 ± 69.95	880.83 ± 70.28
	Mulberry milk 10 g	1085.22 ± 94.28 *p* value = 0.826	745.62 ± 50.82 *p* value = 0.160	623.06 ± 34.05 *p* value = 0.001^∗∗^

Picture updating (1 back)				
Accuracy (%)	Placebo	50.45 ± 4.62	50.00 ± 4.88	53.33 ± 4.24
	Mulberry milk 10 g	58.44 ± 3.89 *p* value = 0.189	54.36 ± 2.68 *p* value = 0.399	52.27 ± 3.91 *p* value = 0.854
Time response (ms)	Placebo	854.69 ± 71.93	809.44 ± 76.16	768.98 ± 66.21
	Mulberry milk 10 g	886.88 ± 84.54 *p* value = 0.773	728.09 ± 41.47 *p* value = 0.314	738.61 ± 55.53 *p* value = 0.725

Picture updating (2 back)				
Accuracy (%)	Placebo	25.33 ± 3.56	30.29 ± 3.32	35.28 ± 3.48
	Mulberry milk 10 g	31.11 ± 4.05 *p* value = 0.287	36.06 ± 3.07 *p* value = 0.247	29.73 ± 3.52 *p* value = 0.268
Response time (ms)	Placebo	825.64 ± 85.22	1005.30 ± 99.37	937.28 ± 69.35
	Mulberry milk 10 g	926.35 ± 69.67 *p* value = 0.363	823.76 ± 60.82 *p* value = 0.112	775.51 ± 70.86 *p* value = 0.107

Flanker arrow				
Accuracy (%)	Placebo	89.61 ± 1.46	85.75 ± 2.00	85.64 ± 1.72
	Mulberry milk 10 g	89.12 ± 1.79 *p* value = 0.832	85.27 ± 1.38 *p* value = 0.844	84.12 ± 1.92 *p* value = 0.559
Time response (ms)	Placebo	928.47 ± 27.67	866.36 ± 25.09	841.54 ± 26.65
	Mulberry milk 10 g	919.88 ± 24.11 *p* value = 0.816	853.81 ± 18.98 *p* value = 0.704	852.91 ± 25.41 *p* value = 0.758

Left right				
Accuracy (%)	Placebo	80.30 ± 2.37	81.70 ± 2.54	80.43 ± 2.41
	Mulberry milk 10 g	82.92 ± 2.43 *p* value = 0.442	76.72 ± 2.19 *p* value = 0.178	74.97 ± 3.00 *p* value = 0.160
Response time (ms)	Placebo	875.67 ± 28.72	780.45 ± 27.15	782.51 ± 23.08
	Mulberry milk 10 g	831.99 ± 29.80 *p* value = 0.294	753.11 ± 16.33 *p* value = 0.373	751.03 ± 23.48 *p* value = 0.342

Up down				
Accuracy (%)	Placebo	84.61 ± 1.62	81.87 ± 2.38	81.99 ± 2.33
	Mulberry milk 10 g	84.80 ± 2.18 *p* value = 0.942	81.47 ± 1.70 *p* value = 0.894	80.45 ± 2.51 *p* value = 0.654
Response time (ms)	Placebo	873.76 ± 27.28	817.67 ± 25.56	774.68 ± 27.15
	Mulberry milk 10 g	874.25 ± 25.72 *p* value = 0.990	783.13 ± 20.03 *p* value = 0.319	774.61 ± 28.53 *p* value = 0.999

Switch-up-Down-left-right				
Accuracy (%)	Placebo	54.27 ± 1.93	57.86 ± 2.34	56.74 ± 2.38
	Mulberry milk 10 g	56.95 ± 2.29 *p* value = 0.373	57.64 ± 1.78 *p* value = 0.943	57.70 ± 2.55 *p* value = 0.784
Time response (ms)	Placebo	960.92 ± 32.96	882.65 ± 36.81	881.97 ± 32.34
	Mulberry milk 10 g	1007.87 ± 30.23 *p* value = 0.297	896.70 ± 23.65 *p* value = 0.744	847.71 ± 32.81 *p* value = 0.459

Odd even				
Accuracy (%)	Placebo	60.66 ± 2.44	62.13 ± 3.15	59.94 ± 3.09
	Mulberry milk 10 g	63.91 ± 2.69 *p* value = 0.374	60.68 ± 1.93 *p* value = 0.685	59.89 ± 2.80 *p* value = 0.991
Time response (ms)	Placebo	1031.75 ± 27.65	946.92 ± 38.70	912.10 ± 31.65
	Mulberry milk 10 g	1064.92 ± 29.65 *p* value = 0.415	948.68 ± 20.69 *p* value = 0.965	914.12 ± 28.93 *p* value = 0.963

Vowel consonant				
Response time (%)	Placebo	66.93 ± 3.10	70.18 ± 2.69	72.27 ± 2.44
	Mulberry milk 10 g	74.12 ± 2.74 *p* value = 0.086	70.51 ± 2.05 *p* value = 0.927	68.82 ± 2.76 *p* value = 0.354
Response time (ms)	Placebo	923.82 ± 25.57	876.37 ± 34.53	867.03 ± 26.78
	Mulberry milk 10 g	962.77 ± 24.11 *p* value = 0.271	886.16 ± 18.96 *p* value = 0.789	863.04 ± 27.90 *p* value = 0.918

Switch letter number				
Accuracy (%)	Placebo	53.77 ± 1.57	57.17 ± 2.00	55.17 ± 2.16
	Mulberry milk 10 g	58.20 ± 1.48 *p* value = 0.052	57.64 ± 1.64 *p* value = 0.864	57.22 ± 2.23 *p* value = 0.512
Response time (ms)	Placebo	961.05 ± 32.88	897.75 ± 43.85	901.76 ± 37.67
	Mulberry milk 10 g	1037.21 ± 31.11 *p* value = 0.096	933.90 ± 23.01 *p* value = 0.425	909.30 ± 31.74 *p* value = 0.878

Stroop Thai				
Accuracy (%)	Placebo	79.50 ± 2.38	82.18 ± 1.59	83.77 ± 1.38
	Mulberry milk 10 g	79.47 ± 2.52 *p* value = 0.993	81.61 ± 1.60 *p* value = 0.825	82.21 ± 1.83 *p* value = 0.501
Time response (ms)	Placebo	777.07 ± 38.57	715.65 ± 37.74	680.24 ± 38.54
	Mulberry milk 10 g	784.76 ± 38.54 *p* value = 0.888	696.70 ± 25.24 *p* value = 0.676	667.03 ± 35.29 *p* value = 0.801

**Table 4:** The effect of mulberry milk on cortisol level and the activities of AChE and MAO in saliva (*n* = 46/group). ^∗^*p* value < 0.05 and ^∗∗∗^*p* value < 0.001, respectively, compared to the placebo group.

Time	Group	Cortisol (ng/mL)	AChE activity (nmol/mg protein)	MAO activity (*μ*mol/h/mg protein)
Baseline	Placebo	4.65 ± 0.35	0.13 ± 0.01	0.06 ± 0.00
	Mulberry Milk 10 g	4.68 ± 0.35 *p* value = 0.95	0.14 ± 0.01 *p* value = 0.39	0.06 ± 0.00 *p* value = 0.76

*T* 1.5 h	Placebo	4.90 ± 0.28	0.13 ± 0.01	0.06 ± 0.00
	Mulberry Milk 10 g	3.47 ± 0.27 *p* value = 0.000^∗∗∗^	0.13 ± 0.01 *p* value = 0.44	0.06 ± 0.00 *p* value = 0.49

*T* 3 h	Placebo	3.90 ± 0.27	0.13 ± 0.01	0.06 ± 0.00
	Mulberry Milk 10 g	3.14 ± 0.21 *p* value = 0.03^∗^	0.14 ± 0.01 *p* value = 0.16	0.06 ± 0.00 *p* value = 0.07

## Data Availability

The authors confirm that data are available and will be provided on request because during this period, all data are in the process of petty patent registration.
